# Coupling life cycle assessment and global sensitivity analysis to evaluate the uncertainty and key processes associated with carbon footprint of rice production in Eastern China

**DOI:** 10.3389/fpls.2022.990105

**Published:** 2022-10-20

**Authors:** Qiang Xu, Jingyong Li, Hao Liang, Zhao Ding, Xinrui Shi, Yinglong Chen, Zhi Dou, Qigen Dai, Hui Gao

**Affiliations:** ^1^ Jiangsu Key Laboratory of Crop Genetics and Physiology/Jiangsu Key Laboratory of Crop Cultivation and Physiology, Agricultural College of Yangzhou University, Yangzhou, China; ^2^ Jiangsu Co-Innovation Center for Modern Production Technology of Grain Crops, Yangzhou University, Yangzhou, China; ^3^ Research Institute of Rice Industrial Engineering Technology of Yangzhou University, Yangzhou, China; ^4^ College of Agricultural Engineering, Hohai University, Nanjing, China; ^5^ Key Laboratory of Crop Harvesting Equipment Technology of Zhejiang Province, Mechanical & Electrical Engineering College of Jinhua Polytechnic, Jinhua, China; ^6^ College of Agriculture, Shanxi Agricultural University, Taigu, China

**Keywords:** carbon footprint, global sensitivity analysis, morris sensitivity analysis, sobol’ sensitivity analysis, uncertainty analysis, life cycle assessment

## Abstract

An accurate and objective evaluation of the carbon footprint of rice production is crucial for mitigating greenhouse gas (GHG) emissions from global food production. Sensitivity and uncertainty analysis of the carbon footprint evaluation model can help improve the efficiency and credibility of the evaluation. In this study, we combined a farm-scaled model consisting of widely used carbon footprint evaluation methods with a typical East Asian rice production system comprising two fertilization strategies. Furthermore, we used Morris and Sobol’ global sensitivity analysis methods to evaluate the sensitivity and uncertainty of the carbon footprint model. Results showed that the carbon footprint evaluation model exhibits a certain nonlinearity, and it is the most sensitive to model parameters related to CH_4_ emission estimation, including *EF_c_* (baseline emission factor for continuously flooded fields without organic amendments), *SF_w_* (scaling factor to account for the differences in water regime during the cultivation period), and *t* (cultivation period of rice), but is not sensitive to activity data and its emission factors. The main sensitivity parameters of the model obtained using the two global sensitivity methods were essentially identical. Uncertainty analysis showed that the carbon footprint of organic rice production was 1271.7 ± 388.5 kg CO_2_eq t^–1^ year^–1^ (95% confidence interval was 663.9–2175.8 kg CO_2_eq t^–1^ year^–1^), which was significantly higher than that of conventional rice production (926.0 ± 213.6 kg CO_2_eq t^–1^ year^–1^, 95% confidence interval 582.5-1429.7 kg CO_2_eq t^–1^ year^–1^) (*p*<0.0001). The carbon footprint for organic rice had a wider range and greater uncertainty, mainly due to the greater impact of CH_4_ emissions (79.8% for organic rice versus 53.8% for conventional rice). *EF_c_*, *t*, *Y*, and *SF_w_* contributed the most to the uncertainty of carbon footprint of the two rice production modes, wherein their correlation coefficients were between 0.34 and 0.55 (*p*<0.01). The analytical framework presented in this study provides insights into future on-farm advice related to GHG mitigation of rice production.

## Graphical Abstract

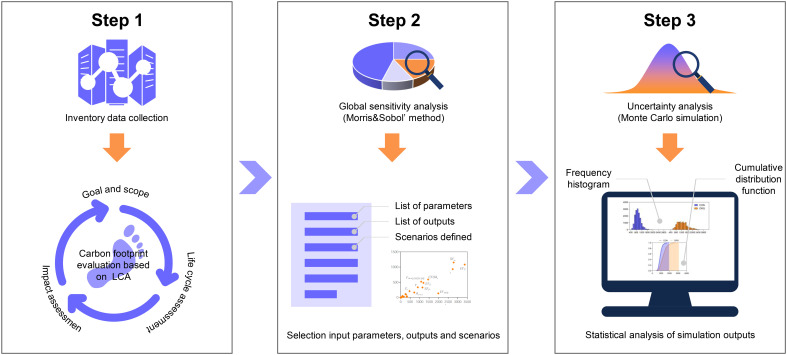

## 1 Introduction

Rice (*Oryza sativa L.*) is a staple food for nearly half of the world’s population, and global rice consumption is projected to increase from 480 million tons (Mt) of milled rice in 2014 to nearly 550 Mt in 2030 (FAO, 2019). Driven by global population and economic growth, global rice production faces the twin challenges of increasing productivity to meet demand and reducing greenhouse gas (GHG) emissions to mitigate climate change (Yuan et al., 2021). Paddy fields are an important source of GHG emissions, accounting for about 50% of total CH_4_ emissions and 10% of total N_2_O emissions from cropland, respectively (Feng et al., 2021a). Carbon footprint evaluation models are important for understanding and quantifying emissions from rice production systems and give a better understanding of emission hotspots and mitigation opportunities. Such models are based on a life cycle assessment approach, the use of which typically varies in target, scope, geographic area, and time span, and can be applied to field scale (Xu Q. et al., 2020; Zhou et al., 2022), regional scale (Chen et al., 2021; Xu Z. et al., 2020), and national or global scale (Lathuillière et al., 2014) GHG assessments. Though such models draw on generic methodologies (IPCC, 2006), the rough methods required for country-level assessments are often insufficient to facilitate detailed policy analysis of the food sector. Consequently, the need for system-level assessments of GHG emissions from food production systems is growing (Singh et al., 2020; Arunrat et al., 2021).

However, the paddy field is complex, and their GHG emissions are strongly influenced by factors such as tillage practices (Xu et al., 2022), fertilization strategies (Liu et al., 2016), and irrigation regimes (Jiang Y. et al., 2019). The existing modelling methods are limited in their ability to accurately capture these complexities, which presents a significant challenge for both modelers and those seeking to leverage such methods for decision-making. Therefore, the carbon emission model of agricultural production system has considerable uncertainty, and it is necessary to conduct sensitivity analysis and uncertainty analysis on the carbon footprint evaluation model, which will help to provide reference for further optimizing parameters and reducing the uncertainty of evaluation results in the future.

Sensitivity analysis can identify high-sensitivity parameters from many input parameters, simplify low-sensitivity parameters, or increase the accuracy of high-sensitivity parameters through more accurate monitoring methods, thereby increasing the overall accuracy of the evaluation model (Reisinger et al., 2017; Jóhannesson et al., 2020). At present, the sensitivity analysis of the carbon footprint evaluation model mainly adopts the local sensitivity method, which only changes one input parameter at a time and evaluates the impact of a single parameter change on the evaluation results. For example, a previous study explored the impact of changes in input parameters on output results in an LCA assessment of Spanish red wine production (Meneses et al., 2016). Xu et al. studied the impact of input parameter changes ( ± 40%) on environmental indicators such as global warming, eutrophication, and acidification, with respect to Chinese export and domestic green tea production (Xu et al., 2021). The changes in coal consumption in tea processing stage are the most sensitive to global warming and acidification, while NH_3_ volatilization in tea cultivation stage is the most sensitive to eutrophication. The crops involved in the above studies were mainly derived from drylands, whose GHG emissions are significantly different from those of paddy fields. All the aforementioned studies used the local sensitivity method, which ignores the nonlinearity of the carbon footprint evaluation model to a certain extent, as well as the influence of the interaction between parameters on the output results. The global sensitivity method comprehensively considers the influence of each parameter and the interaction between parameters on the output result. The commonly used global sensitivity methods include Morris, EFAST, and Sobol’ method (Saltelli et al., 2008). Currently, there is no report on the global sensitivity analysis of the carbon footprint of rice production, and it is unknown whether the sensitivity of the model parameters will be affected by management strategies.

A model is a description of a real system in nature after a series of assumptions and generalizations, which will inevitably lead to a certain amount of uncertainty. Uncertain factors such as monitoring errors, lack of key data, insufficient data representation, selection of accounting models, and allocation methods are inevitable in the carbon footprint evaluation process (De Koning et al., 2010). The uncertainty of carbon footprint assessment results often comes from the uncertainty of input parameters, including activity data, emission factors, model parameters, and scenario selection, which are propagated through the model and lead to uncertainty in estimated values (Milne et al., 2014). In ecological footprint evaluation studies lacking uncertainty analysis, the evaluation results are often questioned and are unconvincing in the interpretation stage (Zhuo et al., 2014; Zhang et al., 2021). Therefore, evaluation and quantification of the uncertainty of input parameters on the results are essential for the analysis of carbon footprint evaluation results. They can help researchers choose more reliable data sources and effectively optimize the data collection plan, and consequently, promote the application and development of product carbon footprint evaluation methods.

Given the impact of uncertainty in interpreting model outputs, as well as the importance of rice production to GHG budgets on a global scale, in this study, we aimed to identify the causes and uncertainties in modelling the farm-scaled GHG footprint affecting rice production in China. We developed a global sensitivity and uncertainty analysis method for system-scaled carbon footprint evaluation and application to rice production with different fertilization strategies, in order to achieve the following: 1) determine the most sensitive parameter in the carbon footprint evaluation of rice production, and improve the evaluation efficiency and accuracy of the model; 2) improve the robustness of the evaluation results and determine the reliability of the evaluation results through uncertainty analysis, providing technical support for further optimizing parameters and reducing the uncertainty of evaluation results in the future.

## 2 Materials and methods

### 2.1 Carbon footprint evaluation methodology

#### 2.1.1 System boundary and functional unit

The carbon footprint was calculated by following the methodology from the IPCC Guidelines for National Greenhouse Gas Inventories (Hergoualc’h et al., 2019). The system boundary of life-cycle product carbon footprint was from cradle to farm gate, including indirect greenhouse gas (GHG) emissions from the production, transportation, and use of agricultural inputs, as well as direct GHG emissions from the farming stage. The functional unit was expressed as kg CO_2_eq t^−1^ year^−1^ rice product (dry matter).

#### 2.1.2 GHG emission calculation from upstream stage

Based on Hergoualc’h et al. (2019), the GHG emissions from upstream stage was calculated by Equation (1).


(1) $$GHG_{raw\;material}=\sum_{i=1}^{n}\left(Ai\;\times \;\varphi i\right)$$

where *GHG_raw material_* is the sum of GHG emissions from upstream stage (kg CO_2_eq ha^–1^ year^−1^); *A_i_* refers to the amount of agri-material inputs in the upstream stage per hectare, including the inputs of rice seeds, chemical fertilizers, organic fertilizers, pesticides, herbicides, and fungicides, as well as the consumption of electricity and diesel during irrigation, land preparation, and harvesting (kg ha^–1^ or kWh ha^–1^); and *φ_i_* refers to the carbon emission coefficients of different agricultural materials (kg CO_2_eq·Unit^–1^), which were mainly derived from the Chinese Life Cycle Database (CLCD v0.8; https://efootprint.net/login) and the Swiss Ecoinvent 2.2 database (https://simapro.com/databases/ecoinvent/).

#### 2.1.3 Field GHG emissions

The CH_4_ emissions from rice cultivation were calculated using Equation (2), which is based on Hergoualc’h et al. (2019) (Tier 2).


(2) $$Q_{CH_{4}}=EFi\times t\times A$$

where Q_CH_4__ denotes annual methane emissions from rice cultivation (kg CH_4_ ha^–1^), *EF_i_* denotes adjusted daily emission factor for a particular harvested area (kg CH_4_ ha^–1^ day^–1^), *t* denotes cultivation period of rice (day); and *A* denotes annual harvested area of rice (ha year^–1^).

Emissions from each different region can be calculated by multiplying a baseline default emissions factor with various scaling factors, as shown in Equation (3) (Hergoualc’h et al., 2019).


(3) $$EF_{i}=EF_{C}\times SF_{W}\times SF_{P}\times SF_{O}\times SF_{s,r}$$

where *EF_c_* is the baseline emission factor for continuously flooded fields without organic amendments; *SF_w_* is a scaling factor to account for the differences in water regime during the cultivation period; *SF_p_* is a scaling factor to account for the differences in water regime in the pre-season before the cultivation period; *SF_O_* is a scaling factor that should vary for both type and amount of organic amendment applied and can be calculated by equation (4); and *SF_s,r_* is a scaling factor for soil type, rice cultivar, etc., if available.

The default conversion factor for farmyard manure was calculated by Equation (4) (Hergoualc’h et al., 2019):


(4) $$SF_{o}=(1+{\sum_{i}ROA_{i}\times CFOA_{i})}^{0.59}$$

where *SF_O_* is the scaling factor for both type and amount of organic amendment applied; *ROA_i_* is the application rate of organic amendment *i*, in dry weight for straw and fresh weight for others in tons ha^–1^; and *CFOA_i_* is the conversion factor for organic amendment *i* (in terms of its relative effect, with respect to straw applied shortly before cultivation).

Hergoualc’h et al. (2019) provided an estimation method for N_2_O emission, where the direct emissions of N_2_O are calculated as follows (Tier 1):


(5) $$Q_{N_{2}O\;(Direct)}=\left[\left(F_{SN}+F_{ON}\right)\times EF_{1}+F_{CR}\times EF_{1FR}\right]\times 44/28$$

where $Q_{N_{2}O\;(Direct)}$ is the direct N_2_O emissions (kg N_2_O year^–1^); *F_SN_* is the annual amount of synthetic N fertilizer applied to soils (kg N year^–1^); *F_ON_* is the annual amount of animal manure, compost, sewage sludge and other organic N additions applied to soils (kg N year^–1^); *F_CR_* is the annual amount of N in crop residues (kg N year^–1^); *EF_1_* is the emission factor for N_2_O emissions from N inputs (kg N_2_O-N (kg N input)^–1^); and *EF_1FR_* is the emission factor for N_2_O emissions from N inputs to flooded rice, kg N_2_O-N (kg N input)^–1^.

The formula for estimating indirect N_2_O emissions was as follows (Hergoualc’h et al., 2019):


(6) $$Q_{N_{2}O\;(Indirect)}=\left[\left(F_{SN}\times Frac_{GASF}+F_{ON}\times Frac_{GASM}\right)\right]\times EF_{4}+\left(F_{SN}+F_{ON}+F_{CR}\right)\times Frac_{LEACH-(H)}\times EF_{5}\times 44/28$$

where $Q_{N_{2}O\;(Indirect)}$ denotes the indirect N_2_O emissions (kg N_2_O year^–1^); *Frac_GASF_* is the fraction of synthetic N fertilizer that volatilizes as NH_3_ and NO_x_, kg N volatilized (kg of N applied)^–1^; *Frac_GASM_* is the fraction of applied organic N fertilizer material (*F_ON_*) that volatilizes as NH_3_ and NO_x_, kg N volatilized (kg of N applied or deposited)^–1^; *EF_4_* is the emission factor for N_2_O emissions from atmospheric deposition of N on soils and water surfaces, kg N–N_2_O (kg NH_3_–N+NO_x_–N volatilized)^–1^; *Frac_LEACH-(H)_* is the fraction of all N added to, or mineralized in, managed soils in regions where leaching or runoff occurs that is lost through leaching and runoff, kg N (kg of N additions)^–1^; and *EF_5_* is the emission factor for N_2_O emissions from N leaching and runoff, kg N_2_O–N (kg N leached and runoff)^–1^.

#### 2.1.4 Calculation of carbon footprint

Yield-based carbon footprint (*CF*) is calculated using Equation (7):


(7) $$CF=\left(GHG_{raw\;material}+Q_{CH_{4}}\times 25+\left(Q_{N_{2}O\;(Direct)}+Q_{N_{2}O\;(Indirect)}\right)\times 298\right)/\left(Y/1000\right)$$

where *CF* is the carbon footprint per unit of yield for rice production (kg CO_2_eq t^–1^ year^−1^); *Y* is the yield of rice (kg ha^–1^). 25 and 298 are the global warming potentials of CH_4_ and N_2_O on a 100-year scale (IPCC, 2013).

### 2.2 Sensitivity analysis

#### 2.2.1 Scenarios, data source, and selection of parameters

Sensitivity analysis was performed under two fertilization scenarios: conventional rice production (CON) and organic rice production (ORG) modes. The geographic area is in Jiangsu Province, which represents an important rice-producing area in eastern China. Collection of agricultural input data of CON and ORG was from 15 and 9 farms through survey questionnaire in 2020–2021. The questionnaire on management practices included: (1) chemical fertilizers, pesticide, fungicide, herbicide, farm yard manure, irrigation water, rice seed, electricity and diesel consumption; (2) yield of rice. In the CON mode, chemical fertilizer and pesticides were used to guarantee high productivity and pest control, while in the ORG mode, only farmyard manure was used and the use of pesticides was not permitted. Rice seed and some fuels, such as electricity and diesel, were used for agricultural operations such as land preparation and irrigation for both scenarios. The calculation of CO_2_ emissions from manufacture of agricultural machinery, i.e., tractor, combine harvester, and pump, is often omitted from carbon footprint studies (Yodkhum et al., 2017). These agricultural machines were excluded from the system in this study because of the lack of actual data on each machine’s lifetime and its overall use times. Moreover, the change in soil carbon sequestration was not considered in this study due to missing data.

The parameters included in the sensitivity analysis of the carbon footprint evaluation model were divided into three categories: 1) background parameters (the carbon emission coefficients of different agricultural materials defined in Equation 1), 2) activity data (the amount of agri-material inputs defined in Equation 1), and 3) CH_4_ and N_2_O estimation parameters. The variation in each category parameter and emission factor would be accounted for in the final result, thereby affecting the objectivity of the final evaluation result (Sykes et al., 2019). Parameters were assumed to be independent from each other, and a uniform distribution was assigned to all parameters because initial information on the distribution characteristics was limited. Many of the previous studies have assumed a uniform distribution of data when encountering similar situations (Vanuytrecht et al., 2014; Liang et al., 2017). A total of 29 and 21 input factors for CON and ORG modes were selected, respectively, for sensitivity analysis (Table A and Table B). Lower and upper boundaries of the parameters used to design the sensitivity analysis were set according to expert knowledge and the values recommended by Hergoualc’h et al. (2019). Specifically, the upper and lower limits of the background parameters were taken from the mean value ± 10%; the upper and lower limits of the activity data were based on the maximum and minimum values of the survey data; the upper and lower limits of the CH_4_ and N_2_O estimation parameters were derived from the empirical values provided by Hergoualc’h et al. (2019). The effects of parameter variation on the output of the carbon footprint model were evaluated.

#### 2.2.2 Local sensitivity analysis

Local sensitivity analysis observes the impact of input parameter changes on the output by changing the value of the input parameters one by one, while the remaining parameters remain unchanged. In this study, each input parameter was changed by ±10%, and then the change in carbon footprint was observed, so that the sensitivity (*E_i_*) of each parameter could be obtained, as calculated by Equation (8):


(8) $$E_{i}=\Delta CF/\Delta A_{i}$$

where *E_i_* was the sensitivity of the input parameter *A_i_*; *ΔA_i_* denotes the change rate of the input parameter *A_i_* (%), with the value of ±10%; and *CF* denotes the corresponding rate of change of the evaluation result *CF* when the input variable *A_i_* has a change rate of *ΔA_i_*. The larger the |*E*| is, the more sensitive the evaluation result *CF* is to the input variable *A*.

#### 2.2.3 Global sensitivity analysis

##### 2.2.3.1 Morris method

The Morris method calculates the elementary effect (EE) of each parameter on the selected output. These elementary effects allow effects of all parameters on the same output to be compared. The Morris method uses the following Equation (9) to calculate the degree of influence (*d*) of each input parameter on the output result:


(9) $$d_{i}(x_{i},\ldots ,x_{k},\Delta )=\frac{\left[y(x_{1},\ldots ,x_{i-1},x_{1}+\Delta ,x_{i+1},\ldots ,x_{k})\right]}{\Delta }$$

where *i* is the number of parameters; *y*(*X*) is the output result of the model; and *X*=(*x_1_*,…,*x_k_*) is the k-dimensional parameter input vector. Upon discretizing the parameter value range (*x_imin_*, *x_imax_*), each parameter can only take a value from these *p* values (*x_imin_*,*x_imin_* +1/(*p*-1)×(*x_imax_*-*x_imin_*), *x_imin_* +2/(*p*-1)×(*x_imax_*-*x_imin_*),…,*x_imax_*), where *p* is the parameter level. Due to the randomness of the parameter values of the Morris method, it is easy to cause value errors; thus, *r* repetitions are required, and the total number of runs of the model is *r*(*k*+1) times, where *k* represents the number of parameters. In this study, *k* was 29 and 21 for CON and ORG mode, respectively. The sensitivity of each parameter is measured by the mean (*μ*) and standard deviation (*σ*) of the *r* “elementary effects”. The larger the value of *μ*, the more sensitive the parameter is to the output result. The value of *σ* represents the interaction between parameters, and the larger the value of *σ*, the stronger the interaction between the parameter under investigation and the other parameters.

The Morris sensitivity analysis for input samples were performed with SimLab (2010), and the evaluation of the carbon footprint model output sets was automated with SimLab (2010). The sensitivity analysis was executed by sampling *r* = 10 elementary effects (Morris, 1991). The model finally ran 300 and 220 times in total for CON and ORG mode, respectively.

##### 2.2.3.2 Sobol’ method

Sobol’ method is a variance-based global sensitivity analysis method, which quantitatively evaluates the effect of each input parameter and the interaction between parameters on the output variable by decomposing the variance of the output variable. When there are *m* input parameters to be analyzed, the *D*(*Y*) of the output result is defined as follows:


(10) $$D(y)=\sum_{i}D_{i}+\sum_{1\leq i<j\leq m}D_{ij}+\cdots +D_{1,2,\cdots ,m}$$

where *D_i_* is the variance generated by parameter *i*; *D_ij_* is the variance produced by the interaction of parameters *i* and *j*; *D_ijk_* is the variance resulting from the interaction of parameters *i*, *j*, and *k*; and D_1,2,…,_ *_m_* is the variance produced by the joint interaction of *m* parameters.

For parameter *i*, the first-order sensitivity index (*S_i_*) can be used to reflect the sensitivity of that single parameter, and the full-order sensitivity index (ST*_i_*) can be used to indicate the common effect of that parameter and all other parameters. The equations to calculate these indices are as follows:


(11) $${\text{S}}_{\text{i}}=\frac{{\text{D}}_{\text{i}}}{\text{D}}$$

(12) $$ST_{i}=1-\frac{D_{:i}}{D}$$

where *D_:i_* represents the variances of parameters other than parameter *i*.

The input samples for sensitivity analysis were generated with SimLab (2010), and Sobol’ analysis method was executed using *m* = 29 and 21 input factors for CON and ORG mode, respectively, as well as a sample size of *n*(*m* + 2) model input sets, where *n* is defined as having a range of 100 or higher (Saltelli et al., 2000). In this study, we used *n* = 496 for a total of 15376 and *n* = 490 for a total of 11270 input parameter sets for conventional rice, and organic rice, respectively.

### 2.3 Uncertainty analysis

Uncertainty analysis helps to increase the robustness of evaluation results (Milne et al., 2014; Sykes et al., 2019). Sobol’ analysis embeds a Monte Carlo module in the SimLab (2010) software, which enables the quantification of propagation of the uncertainties in the model inputs through the model. Based on the simulation values of Sobol’ analysis described above, an uncertainty analysis was conducted. Mean, median, standard deviation, minima, maxima, and 2.5% and 97.5% quantiles of carbon footprints with a 95% confidence interval were calculated, and the frequency histogram and the cumulative distribution function were also provided.

### 2.4 Statistical analysis

Statistical analyses were carried out using SPSS 22.0 software, and t-test of least significant difference (LSD) method was used to analyze the differences in carbon footprint between CON and ORG modes, followed by least significant difference (LSD) tests (*p*< 0.05) with a significance level of 5%. Pearson correlation analysis was used between input parameters and carbon footprint (two-tailed).

## 3 Results

### 3.1 Local sensitivity analysis

**Figure 1:**
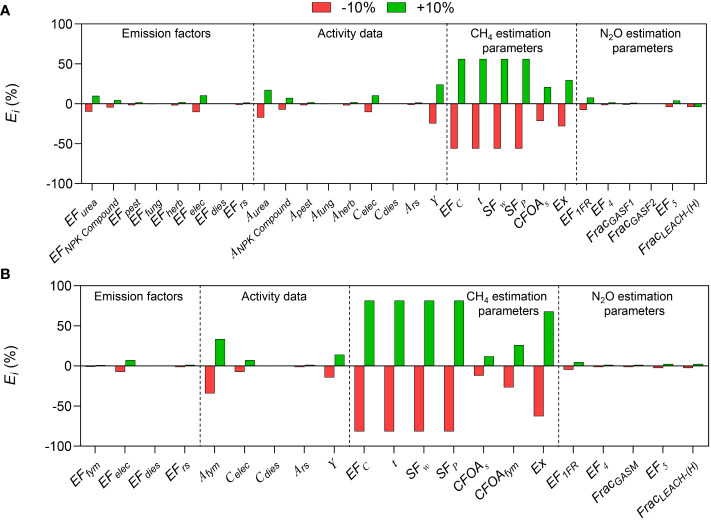
Sensitivity analysis of each parameter of the carbon footprint evaluation model to the carbon footprint by a local sensitivity analysis in **(A)** conventional and **(B)** organic rice production.

The sensitivity (*E_i_*) of each parameter can be obtained by changing each input parameter by ±10% and then observing the change in carbon footprint (**Figure 1**). The impact of CH_4_ emission estimation parameters on carbon footprint is higher than that of emission factors, activity data, and N_2_O emission estimation parameters. For conventional rice production mode, among the CH_4_ emission estimation parameters, *EF_c_*, *t*, *SF_w_*, and *SF_p_* had the greatest effect on carbon footprint (with an *E_i_* of ±56.1%), followed by *E_x_* (with an *E_i_* of ±28.1%), *CFOA_s_* which was ranked the last (with an *E_i_* of ±21.5%). Among activity data, the *E_i_* of *Y*, *A_urea_*, and *C_elec_* was ±24.6%, ± 17.2%, and ±10.4%, respectively; while the *E_i_* of other parameters were lower than ±10%. Emission factors of background database and N_2_O emission estimation parameters had relatively low *E_i_*. The trend was similar in the CON and ORG modes. The above analysis showed that it was impossible to know which parameter was the most sensitive from the local sensitivity analysis alone, as the sensitivities (*E_i_*) of *EF_c_*, *t*, *SF_w_*, and *SF_p_* were consistent.

### 3.2 Global sensitivity analysis

#### 3.2.1 Morris sensitivity analysis

**Figure 2:**
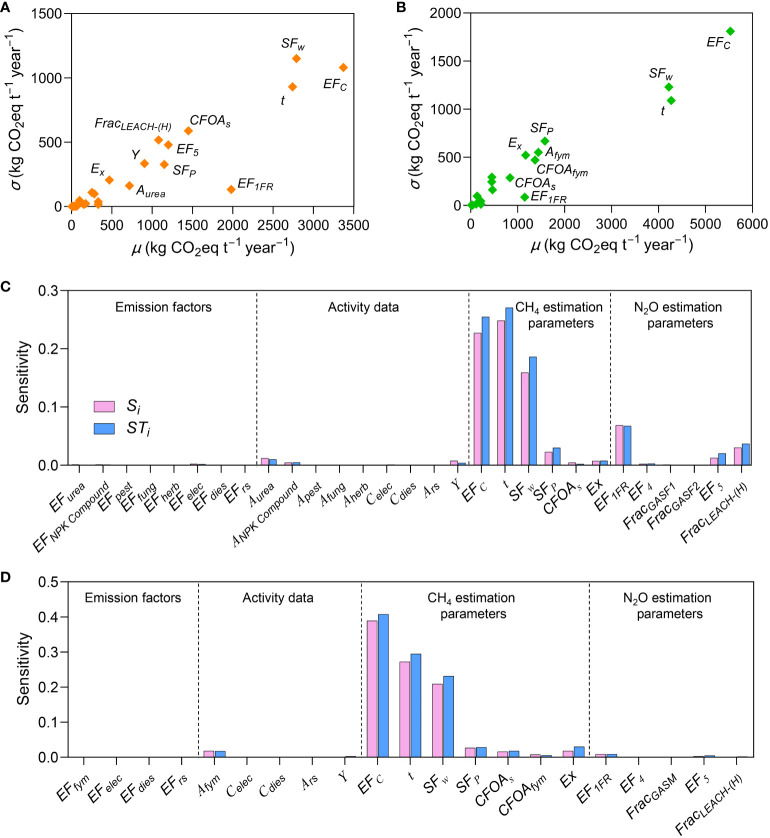
Sensitivity analysis indices of conventional (CON) and organic (ORG) rice production modes, as per global sensitivity analysis. Morris mean effect (*μ*) to the carbon footprint, as per Morris method in **(A)** CON and **(B)** ORG. Sobol’ first-order indices (*S_i_*), and Sobol’ total sensitivity (ST*_i_*) to, the carbon footprint, as per a Sobol’ variance-based method in **(C)** CON and **(D)** ORG.

**Figures 2 A–B** shows the mean (*μ*) and variance (*σ*) of the Morris sensitivity index of the input parameters of the carbon footprint evaluation model to carbon footprint of CON and ORG modes. Overall, the estimation parameters of CH_4_ emission generally showed greater sensitivity than emission factors of background database, activity data, and N_2_O emission estimation parameters. For conventional rice production, the sensitivity of *EF_c_* (*μ*=3370.0 kg CO_2_eq t^–1^ year^−1^, *σ*=1080.0 kg CO_2_eq t^–1^ year^−1^), *SF_w_* (*μ*=2790.0 kg CO_2_eq t^–1^ year^−1^, *σ*=1150.0 kg CO_2_eq t^–1^ year^−1^), and *t* (*μ*=2740.0 kg CO_2_eq t^–1^ year^−1^, *σ*=930.0 kg CO_2_eq t^–1^ year^−1^) to carbon footprint was high among the sensitivities of CH_4_ emission estimation parameters. A higher σ indicates that these three parameters interact strongly with other parameters. *CFOA_s_*, *EF_5_*, and *Frac_LEACH-(H)_* had relatively high sensitivity to carbon footprint, and thus, are relevant to the estimation of CH_4_ and N_2_O emissions. Notably, although *EF_1FR_* had a relatively high *μ* (1980.0 kg CO_2_eq t^–1^ year^−1^), its low *σ* (132.7 kg CO_2_eq t^–1^ year^−1^) indicated weak interaction with other parameters. For organic rice production mode, the sensitivities of *EF_c_*, *SF_w_*, and *t* to carbon footprint were also high, among the sensitivities of various parameters. The difference is that the *μ* and *σ* of these three parameters in the ORG mode were higher than those in the CON mode, indicating that CH_4_ emissions have a greater impact on the carbon footprint of the ORG mode.

#### 3.2.2 Sobol’ sensitivity analysis

The Sobol’ first-order indices (*S_i_*) and Sobol’ total sensitivity (ST*_i_*) as per Sobol’ global sensitivity analysis, are shown in **Figure 2C–D**. Similar to the results of the Morris analysis, the sensitivity indices of CH_4_ emission estimation parameters were the highest among those of all parameters associated with Sobol’ sensitivity analysis. *EF_c_*, *SF_w_*, and *t* collectively explained 15.9%−27.0% and 20.9%−40.8% of the variability of carbon footprint for CON and ORG, respectively. The difference between ST*_i_* and *S_i_* of a parameter is the degree of influence of the interaction between that parameter and other parameters on the output results of the carbon footprint evaluation model. The larger the difference, the stronger the interaction of the parameter. *EF_c_*, *SF_w_*, and *t* indicated greater interaction with other parameters, which corresponded to the *σ* values noted as per the Morris analysis method. Overall, the results of the two global sensitivity analyses were consistent, and the results show that the CH_4_ and N_2_O emission estimation parameters were more sensitive to the carbon footprint of CON mode, whereas only the CH_4_ emission estimation parameters were more sensitive to the carbon footprint of ORG mode.

#### 3.2.3 Comparison between Morris and Sobol’ method

Two methods were used for the global sensitivity analysis; correlations between the full-order sensitivity index (ST*_i_*) values calculated as per Sobol’ method and the *μ* values calculated as per Morris method for CON and ORG modes are shown in **Figure S1**. The two methods yielded *r* values of 0.90 for CON and 0.96 for ORG, indicating a very high correlation between the two methods used.

### 3.3 Uncertainty analysis

**Figure 3:**
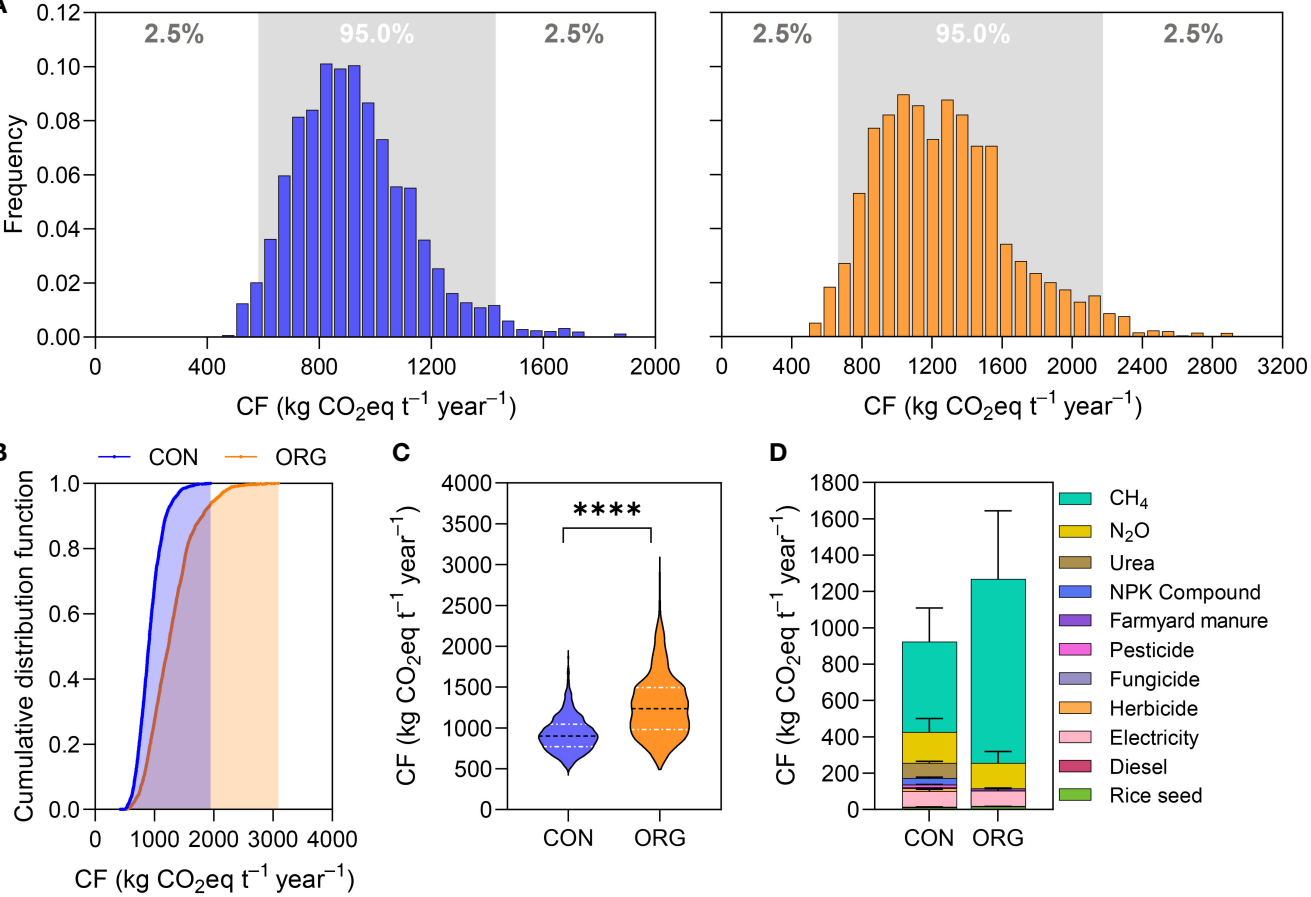
Uncertainty analysis of carbon footprint (CF) of conventional (CON) and organic (ORG) rice production. **(A)** Frequency histogram of CON (left) and ORG (right); **(B)** cumulative distribution function; **(C)** violin plot of carbon footprint based on Sobol’ variance-based method. **** indicates significant difference at *p*<0.0001 level by a t-test of least significant difference (LSD) method. **(D)** Breakdown of the total CF (calculated based on Sobol’ variance-based method) to the level of individual emissions sources of CON and ORG. Error bars indicate 5-95% confidence interval (CI) for each source, calculated *via* Monte Carlo simulation.

Uncertainty analysis can be used to determine the correlation between input parameters and output results, which helps increase the robustness of results during the interpretation stage in LCA studies. Thus, we conducted an uncertainty analysis based on 15360 outputs of CON and 11264 outputs of ORG using Sobol’ analysis. **Figure 3A** shows the frequency histogram of simulation outputs of CON and ORG modes. The distributions of carbon footprints of both modes were positively skewed, with skewness of 0.82 and 0.78 for CON and ORG, respectively. Simulated carbon footprint was found to be higher for the ORG mode than the CON mode, as indicated by a steeper line for the former (**Figure 3B**). **Figure 3C** shows a violin plot of the simulated carbon footprint of the two rice production modes. The mean value was 926.0 ± 213.6 kg CO_2_eq t^–1^ year^−1^ for CON mode and 1271.7 ± 388.5 kg CO_2_eq t^–1^ year^−1^ for ORG mode, and the difference reached a significant level (*p*<0.0001) (**Table 1**). The range of carbon footprints for CON and ORG modes was 424.6–1946.9 kg CO_2_eq t^–1^ year^−1^ and 491.6–3092.8 kg CO_2_eq t^–1^ year^−1^, respectively. The 25% percentile and 75% percentile of CON mode were 772.8 kg CO_2_eq t^–1^ year^−1^ and 1044.3 kg CO_2_eq t^–1^ year^−1^, respectively, while those of ORG mode were 979.6 kg CO_2_eq t^–1^ year^−1^ and 1495.8 kg CO_2_eq t^–1^ year^−1^, respectively. The CON mode exhibited 5% and 95% CIs of 582.5 and 1429.7 kg CO_2_eq t^–1^ year^−1^, respectively, whereas the ORG mode exhibited 5% and 95% CIs of 663.9 and 2175.8 kg CO_2_eq t^–1^ year^−1^, respectively. The reason for ORG mode showing a broader range and greater uncertainty of carbon footprint results might be that it was affected by emissions of CH_4_ to a greater degree. Breakdown of the system emissions into different sources is shown in **Figure 3D**. The emissions of CH_4_ accounted for as high as 53.8% and 79.8% for the CON and ORG mode, respectively. However, the emissions of N_2_O and embedded carbon emission in the production of upstream agri-materials accounted for 18.5% and 27.6% for CON mode, respectively, and 11.0% and 9.1% for ORG mode, respectively.

**Table 1:** Uncertainty analysis statistics of all output responses of conventional and organic rice production modes, as determined using Sobol’ global sensitivity analysis simulations.

Item	Unit	Conventional rice	Organic rice
No. of values	–	15376	11270
Minimum	kg CO_2_eq·t^−1^ year^−1^	424.6	491.6
25% Percentile	kg CO_2_eq·t^−1^ year^−1^	772.8	979.6
Median	kg CO_2_eq·t^−1^ year^−1^	902.2	1235.3
75% Percentile	kg CO_2_eq·t^−1^ year^−1^	1044.3	1495.8
Maximum	kg CO_2_eq·t^−1^ year^−1^	1946.9	3092.8
Mean	kg CO_2_eq·t^−1^ year^−1^	926.0	1271.7
S.D.	kg CO_2_eq·t^−1^ year^−1^	213.6	388.5
Skewness	–	0.82	0.78
Kurtosis	–	1.12	0.71
5% CI	kg CO_2_eq·t^−1^ year^−1^	582.5	663.9
95% CI	kg CO_2_eq·t^−1^ year^−1^	1429.7	2175.8

S.D., standard deviation; CI, confidence interval.

**Figure 4:**
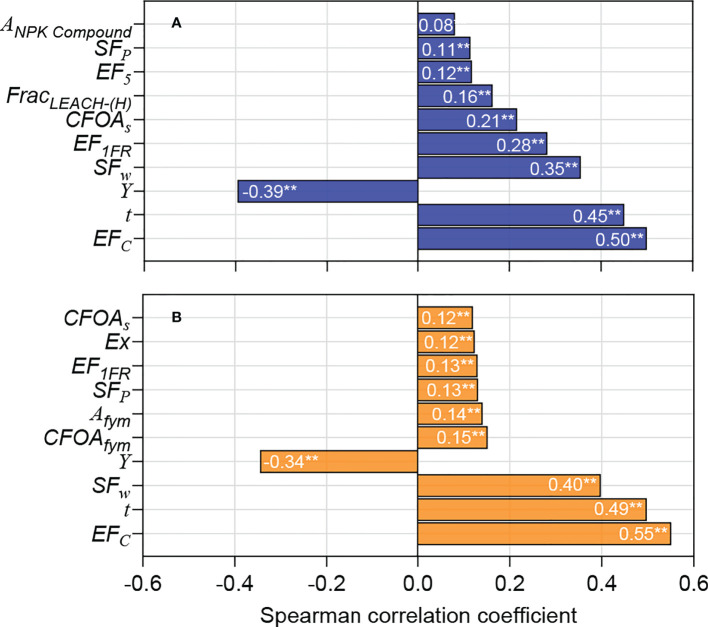
Tornado graphs showing the model inputs that according to the Pearson Rank correlation coefficient (two-tailed test), affected the uncertainty in estimated carbon footprint the most for **(A)** conventional and **(B)** organic rice production. ** indicates significant difference at *p*<0.01 level.

We ranked the top 10 input parameters with greater uncertainty affecting the carbon footprint according to the correlation coefficient (**Figure 4**). In addition to the CH_4_ estimation parameters, the system output (*Y*) also contributed significantly to the uncertainty of the carbon footprint. Specifically, taking conventional rice as an example, *EF_c_* had the largest impact on the uncertainty of the carbon footprint, with a correlation coefficient *r* of 0.50**; the correlation reached an extremely significant level (*p*<0.01). The next most influential inputs were *t* (*r*=0.45**), *Y* (*r*=–0.39**), and *SF_w_* (*r*=0.35**). Several parameters related to N_2_O emission estimation (*EF_1FR_*, *Frac_LEACH-(H)_*, and *EF_5_*) had relatively larger impacts on the uncertainty of carbon footprint, while others had relatively smaller impacts. Similarly, for organic rice, the most influential inputs were *EF_c_* (*r*=0.55**), *t* (*r*=0.49**), *SF_w_* (*r*=–0.40**), and *Y* (*r*=–0.34**); the correlation reached an extremely significant level (*p*<0.01). However, other parameters had relatively smaller impacts on the uncertainty of the carbon footprint, with correlation coefficient *r* not being more than 0.15.

## 4 Discussion

The current study involved a novel approach to identify and quantify the sources and effects of sensitivity and uncertainty in the carbon footprint of rice production, based on IPCC guidelines. Through the comparison of local sensitivity analysis and global sensitivity analysis of carbon footprint evaluation of rice production, in this study, we found that although the local sensitivity analysis identifies the more sensitive input parameters, the nonlinearity of the evaluation model cannot be considered satisfactory. It is impossible to determine which parameter is the most sensitive. From **Figures S2–S3**, it is clear that the carbon footprints of CON and ORG modes had extremely significant positive correlations with CH_4_ emission, wherein the correlation coefficient *r* reached 0.79 and 0.91, respectively. However, there is no reliable linear correlation between the parameters (*EF_c_*, *SF_w_*, and *t*) involved in the estimation of CH_4_ emissions and carbon footprint. This suggests that the carbon footprint model exhibits a certain amount of nonlinearity, and the two global sensitivity analyses used in this study (Morris and Sobol’ method) account for the interaction among the input parameters.

The results of this study showed that the contribution of CH_4_ emissions to the carbon footprint of CON and ORG modes was as high as 53.8% and 79.8%, respectively. This result is concordant with those of previous studies (Jiang Z. et al., 2019; Arunrat et al., 2021; Kashyap and Agarwal, 2021). Therefore, the uncertainty in the carbon footprint mainly stemmed from the uncertainty in the estimation of CH_4_ emissions. CH_4_ emissions from paddy field ecosystems were affected by many factors, such as soil properties, climatic factors, water and nutrient management strategies, organic substitutions, and rice varieties, with a great degree of variability (Feng et al., 2021; Yang et al., 2021). In the process of carbon footprint evaluation, when the field measurement conditions for CH_4_ are not available, notable attention must be paid to the values of *EF_c_*, *SF_w_*, and *t*. The IPCC guidelines provide an estimate of the daily time step CH_4_ emissions from paddy fields, and the emission factor in the model can be modified according to specific water and fertilizer management or organic substitutions. *SF_w_* is a scaling factor that accounts for the differences in water regime during the cultivation period. CH_4_ emission from paddy fields is the net effect of CH_4_ generation, oxidation, and transport in soil. Water status of soil strongly affects the redox environment of paddy soil (Qian et al., 2022). Therefore, *SF_w_* has a remarkable influence on the estimation of CH_4_ emissions from paddy fields. The rice cultivation period (*t*) is strongly influenced by the rice variety (Feng et al., 2021; Song et al., 2022). According to the sowing period, growth period, and maturity period, paddy can be divided into three categories: early paddy, middle paddy, and late paddy. Generally, the growth period of early paddy is 90-120 days, middle paddy is 120-150 days, and late paddy is 150-170 days (Wang, 2016). Due to the different climatic conditions in different regions, the cultivation period of the same rice variety in different regions is different. It can be seen that the variability of the parameter *t* is extremely large, which has a notable impact on the uncertainty of CH_4_ emission estimation and carbon footprint evaluation results. The correlation analysis showed that the system output *Y* is significantly negatively correlated with the carbon footprint (*p*<0.01), and this parameter had a notable impact on the uncertainty of the carbon footprint (**Figure 4**). The value of *Y* is mainly obtained from investigation or on-field measurement; thus, it is necessary to exercise caution when collecting the data. For the accurate values of the above coefficients, analysts require extensive experience in agronomy and ecology; this is crucial for increasing the credibility of the evaluation results. Compared with the estimation parameters for CH_4_ emission, the parameters related to the estimation of N_2_O emission had lesser influence on the uncertainty of carbon footprint. Among all the parameters, *EF_1FR_* had the greatest impact on carbon footprint. This parameter is frequently used in carbon footprint studies of crop production to quantify N_2_O emissions from rice fields due to nitrogen inputs (Yodkhum et al., 2017). In the present study, the impact of *EF_1FR_* on the carbon footprint of CON mode was greater compared to that of ORG model, owing to the higher exogenous nitrogen input for CON mode than that for ORG mode. Activity data and their carbon emission factors had a lower impact on the carbon footprint than that of parameters related to the estimation of CH_4_ and N_2_O emission. Among them, fertilizer input, electricity consumption, and system output (rice yield) had a greater impact on the carbon footprint, indicating that these coefficients should be given priority when collecting activity data.

According to the above analysis, adopting more accurate inventory data will effectively reduce the uncertainty of the evaluation results. Therefore, this study proposes the following inventory data collection optimization scheme. Factors related to greenhouse gas (especially for CH_4_) estimates should be given priority, followed by activity data and their emission factors. System-scale carbon footprint evaluation should use field-measured CH_4_ and N_2_O emission levels as much as possible because these values correspond to specific rice varieties, management practices, and soil-climate conditions. In cases where measured values cannot be provided, experts should be consulted to determine the parameters involved in the estimation of GHG emissions. The fundamental requirement is that the basic research on the mechanism of GHG emissions from paddy fields should be strengthened, which is crucial for improving the modelling of GHG emissions. Therefore, with the continuous improvement and development of the basic database of carbon footprint evaluation in the agricultural field, the relevant carbon emission parameters will have better temporal representation, statistical representation, geographical representation, data sources, and technical representation.

The sensitivity and uncertainty analyses of the carbon footprint of rice production conducted in the current study provides a research framework that is applicable to crops in other countries or regions. We strongly recommend that emission factors or model parameters derived from local soil-climate conditions be prioritized when other regions use the analytical framework of this study. The limitations of this study are as follows. 1) In global sensitivity analysis, parameters were assumed to be independent from each other. However, in this study, there were some correlations between parameters, such as fertilizer application amount and rice yield. The global sensitivity analysis algorithm will not work if the parameters vary randomly and there is a correlation between the parameters. This is a shortcoming of this study. In future research, the parameters need to be independent of each other and the algorithm needs to be further improved, which will help to improve the credibility of the evaluation results. 2) The fertilizers in the activity data in this study are the commonly used urea and farmyard manures. The impact of activity data and their emission factors on carbon footprint uncertainty may change when new fertilizers such as nitrification inhibitors and biochar-based fertilizers are used. 3) The results of this study suggested that different fertilization scenarios have a large impact on carbon footprint uncertainty. However, this study did not consider scenarios such as different rice varieties, irrigation regimes, and tillage practices, which should be addressed in future studies.

## 5 Conclusions

This study provided a general approach to the sensitivity and uncertainty analysis of a system-scaled carbon footprint evaluation model for crop production, which is essential for objective, accurate, and efficient accounting of GHG emissions from crop production. Local sensitivity analysis cannot consider the nonlinearity of the carbon footprint evaluation model, while global sensitivity analysis overcomes this problem and effectively identifies the key parameters of the model. The carbon footprint evaluation model was most sensitive to model parameters related to CH_4_ emission (primarily *EF_c_*, *SF_w_*, and *t*), while it was relatively insensitive to N_2_O emission estimation parameters, activity data, and its emission factors. The main model sensitivity parameters of Morris and Sobol’ global sensitivity analysis methods were essentially the same. Uncertainty analysis results based on the Sobol’ method showed that the carbon footprint of CON mode was 926.0 ± 213.6 kg CO_2_eq t^–1^ year^–1^ with 95% confidence interval (582.5, 1429.7), and the carbon footprint of ORG mode is 1271.7 ± 388.5 kg CO_2_eq t^–1^ year^–1^ with 95% confidence interval (663.9, 2175.8); the latter is extremely significantly higher than the former (*p*<0.0001). *EF_c_*, *t*, *Y*, and *SF_w_* contributed the most to the uncertainty of the carbon footprint of both rice production modes, and the correlation coefficient *r* was between 0.34 and 0.55; the *r* values for these parameters were very significant (*p*<0.01). Certainty can effectively improve the robustness and credibility of evaluation results. Reducing the uncertainty of these coefficients can effectively improve the robustness and credibility of the evaluation results. The analytical framework developed in the current study is applicable to other crops in different regions, and it can be used to guide researchers to formulate data inventory collection and optimization plans for carbon footprint evaluation of crop production, as well as to help policy makers assess whether the GHG mitigation is significant in future grain production.

## Data availability statement

The original contributions presented in the study are included in the article/**Supplementary Material**. Further inquiries can be directed to the corresponding author.

## Author contributions

QX: Conceptualization, methodology, formal analysis, writing - original draft, writing - review & editing, funding acquisition. JL: Visualization, data curation. HL: Methodology. ZDi: Investigation. XS: Methodology. YC: Investigation, data curation. ZDo: Investigation, data curation. QD: Supervision, funding acquisition. HG: Supervision. All authors jointly reviewed the manuscript and approved for publication. All authors contributed to the article and approved the submitted version.

## Funding

The work was supported by the National Key Research and Development Project (2021YFD1700800), the Natural Science Foundation of Jiangsu Province (BK20210791), the Priority Academic Program Development of Jiangsu Higher Education Institutions (PAPD), the Lv Yang Jin Feng Research Plan of Yangzhou City (YZ-LYJF2020PHD100), and the Scientific Research Startup Foundation for Urgently-Needed Talents, Yangzhou University.

## Conflict of interest

The authors declare that the research was conducted in the absence of any commercial or financial relationships that could be construed as a potential conflict of interest.

## Publisher’s note

All claims expressed in this article are solely those of the authors and do not necessarily represent those of their affiliated organizations, or those of the publisher, the editors and the reviewers. Any product that may be evaluated in this article, or claim that may be made by its manufacturer, is not guaranteed or endorsed by the publisher.
