# Supplementary material for: Coupling life cycle assessment and global sensitivity analysis to evaluate the uncertainty and key processes associated with carbon footprint of rice production in Eastern China
Source: Front Plant Sci. 2022 Oct 20;13:990105. doi: 10.3389/fpls.2022.990105 (PMC9632737; doi:10.3389/fpls.2022.990105)
Supplement: Supplementary file 1 [file DataSheet_1.pdf]

Coupling life cycle assessment and global sensitivity analysis to evaluate the uncertainty and key processes associated with carbon footprint of rice production in Eastern China

Qiang Xu [a, b, c], Jingyong Li [c], Hao Liang [d], Zhao Ding [e], Xinrui Shi [f], Yinglong Chen [a, b, c], Zhi Dou [a, b, c], Qigen Dai [a, b, c], Hui Gao [a, b, c *]

[a] Jiangsu Key Laboratory of Crop Genetics and Physiology / Jiangsu Key Laboratory of Crop Cultivation and Physiology, Agricultural College of Yangzhou University, Yangzhou 225009, China
[b] Jiangsu Co-Innovation Center for Modern Production Technology of Grain Crops, Yangzhou University, Yangzhou 225009, China
[c] Research Institute of Rice Industrial Engineering Technology of Yangzhou University, Yangzhou 225009, China
[d] College of Agricultural Engineering, Hohai University, Nanjing 210098, China
[e] Key Laboratory of Crop Harvesting Equipment Technology of Zhejiang Province, Mechanical&Electrical Engineering College of Jinhua Polytechnic, Jinhua 321017, China
[f] College of Agriculture, Shanxi Agricultural University, Taigu 030801, China

Hui Gao* (the corresponding author)

Affiliation: Agricultural College, Yangzhou University
Postal address: Daxue South Road No. 88, Yangzhou City 225009, Jiangsu Province, P.R. China
Phone number: 86-0514-8797-4556
E-mail address: gaohui@yzu.edu.cn

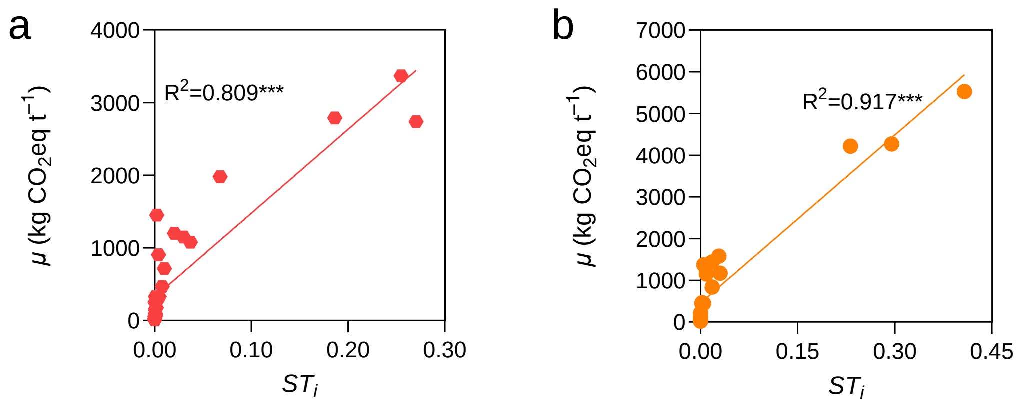

Fig S1. Correlations between the Sobol' method full-order sensitivity index (ST_i) and Morris method μ for conventional (**a**) and organic (**b**) rice production mode.

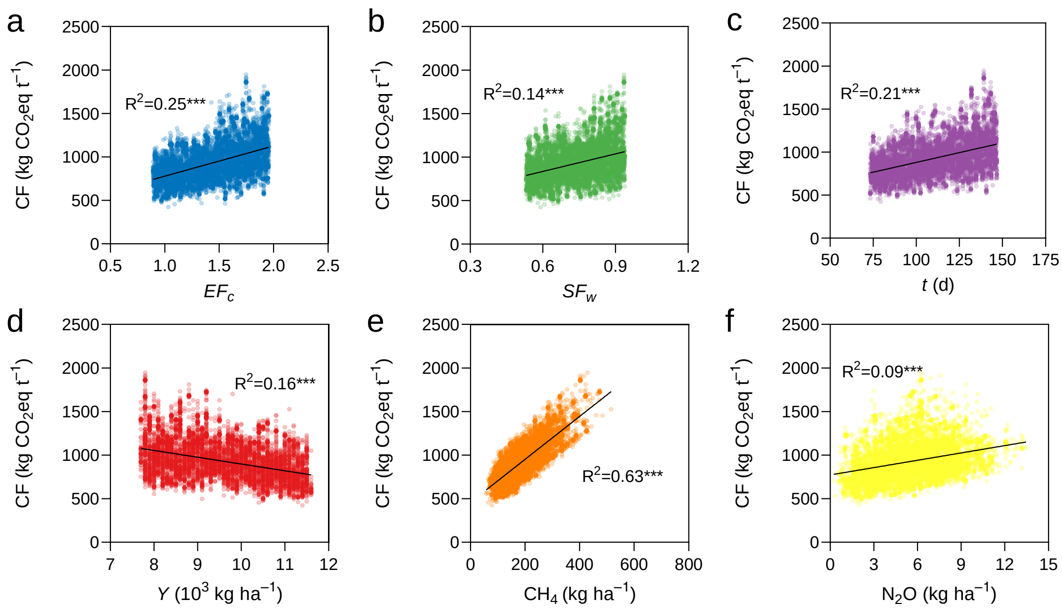

Fig S2. Correlations between carbon footprint (CF) and several parameters, CH_4, and N_2O of conventional rice production. **a-f**, In each panel, the black line indicates the regression fitting results, and the correlation coefficient is labelled in the plot. Panels represent EF_c (**a**), SF_w (**b**), t (**c**), Y (**d**), CH_4 (**e**), N_2O (**f**). ***, **, * Statistically significant at $p < 0.001$, $p < 0.01$, and $p < 0.1$ level, respectively.

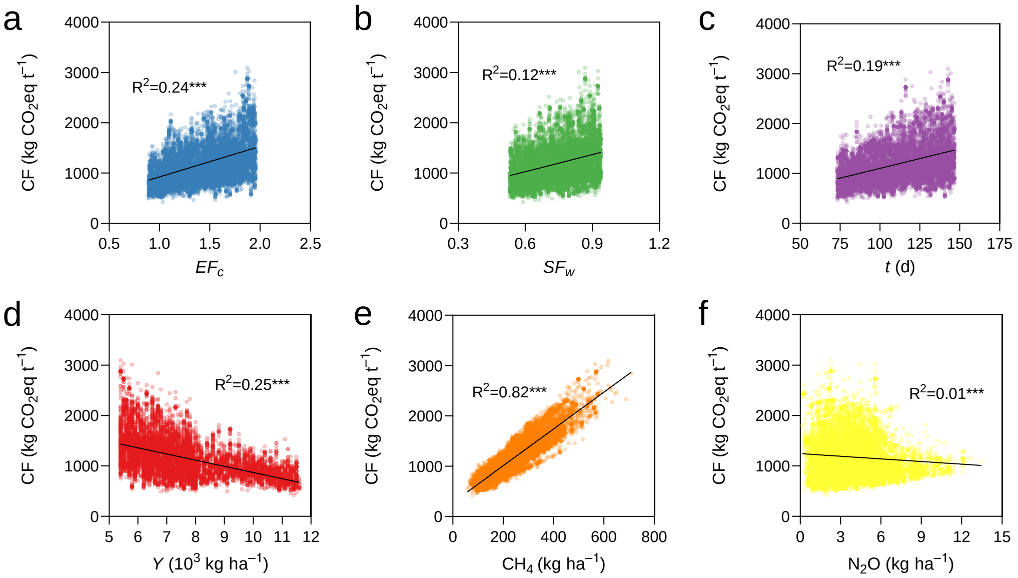

Fig S3. Correlations between carbon footprint (CF) and several parameters, CH_4, and N_2O of organic rice production. **a-f**, In each panel, the black line indicates the regression fitting results, and the correlation coefficient is labelled in the plot. Panels represent EF_c (**a**), SF_w (**b**), t (**c**), Y (**d**), CH_4 (**e**), N_2O (**f**). ***, **, * Statistically significant at $p < 0.001$, $p < 0.01$, and $p < 0.1$ level, respectively.
