# Supplementary material for: Coupling life cycle assessment and global sensitivity analysis to evaluate the uncertainty and key processes associated with carbon footprint of rice production in Eastern China
Source: Front Plant Sci. 2022 Oct 20;13:990105. doi: 10.3389/fpls.2022.990105 (PMC9632737; doi:10.3389/fpls.2022.990105)
Supplement: Supplementary file 2 [file Table_1.docx]

**Appendix**

**Table A** Selection of input parameters of carbon footprint evaluation model of conventional rice production mode.

| Category | Parameters | Description | Lower boundary | Upper boundary | Initial value | Source ^a^ |
| --- | --- | --- | --- | --- | --- | --- |
| Background parameters | *EF_urea_* | Emission factor of urea (kg CO_2_ kg^–1^) | 2.38 | 2.90 | 2.64 | Database |
|  | *EF_NPK Compound_* | Emission factor of NPK compound (kg CO_2_ kg^–1^) | 0.86 | 1.05 | 0.96 | Database |
|  | *EF_pest_* | Emission factor of pesticide (kg CO_2_ kg^–1^) | 11.87 | 14.51 | 13.19 | Database |
|  | *EF_fung_* | Emission factor of fungicide (kg CO_2_ kg^–1^) | 10.59 | 12.94 | 11.77 | Database |
|  | *EF_herb_* | Emission factor of herbicide (kg CO_2_ kg^–1^) | 15.29 | 18.69 | 16.99 | Database |
|  | *EF_elec_* | Emission factor of electricity consumption (kg CO_2_ kWh^–1^) | 1.14 | 1.40 | 1.27 | Database |
|  | *EF_dies_* | Emission factor of diesel consumption (kg CO_2_ kg^–1^) | 0.33 | 0.41 | 0.37 | Database |
|  | *EF_rs_* | Emission factor of rice seed (kg CO_2_ kg^–1^) | 1.69 | 2.07 | 1.88 | Database |
| Activity data | *A_urea_* | Amount of urea (kg ha^–1^) | 240 | 360 | 300 | Investigation |
|  | *A_NPK Compound_* | Amount of NPK Compound (kg ha^–1^) | 300 | 450 | 375 | Investigation |
|  | *A_pest_* | Amount of pesticide (kg ha^–1^) | 8.96 | 13.44 | 11.20 | Investigation |
|  | *A_fung_* | Amount of fungicide (kg ha^–1^) | 2.52 | 3.78 | 3.15 | Investigation |
|  | *A_herb_* | Amount of herbicide (kg ha^–1^) | 7.14 | 10.70 | 8.92 | Investigation |
|  | *C_elec_* | Consumption of electricity (kWh ha^–1^) | 524 | 786 | 655 | Investigation |
|  | *C_dies_* | Consumption of diesel (kg ha^–1^) | 58 | 87 | 72 | Investigation |
|  | *A_rs_* | Amount of rice seed (kg ha^–1^) | 48 | 72 | 60 | Investigation |
|  | *Y* | Yield of rice (kg ha^–1^) | 7711 | 11567 | 9639 | Investigation |
| CH_4_ estimation parameters | *EF_C_* | Baseline emission factor for continuously flooded fields without organic amendments (kg CH_4_ ha^–1^ day^–1^) | 0.89 | 1.96 | 1.32 | IPCC, 2019 |
|  | *t* | Cultivation period of rice (d) | 73 | 147 | 118 | Investigation |
|  | *SF_w_* | Scaling factor to account for the differences in water regime during the cultivation period (–) | 0.53 | 0.94 | 0.71 | IPCC, 2019 |
|  | *SF_P_* | Scaling factor to account for the differences in water regime in the pre-season before the cultivation period (–) | 0.80 | 0.99 | 0.89 | IPCC, 2019 |
|  | *CFOA_s_* | Conversion factor for straw (Straw incorporated >30 days before cultivation) | 0.11 | 0.28 | 0.19 | IPCC, 2019 |
|  | *Ex* | The exponent in Equation 5.3 | 0.54 | 0.64 | 0.59 | IPCC, 2019 |
| N_2_O estimation parameter | *EF_1FR_* | Emission factor for N_2_O emissions from N inputs to flooded rice (single and multiple drainage) (kg N_2_O-N (kg N input)^–1^) | 0 | 0.016 | 0.005 | IPCC, 2019 |
|  | *EF_4_* | Emission factor for N_2_O emissions from atmospheric deposition of N on soils and water surfaces (kg N–N_2_O (kg NH_3_–N + NO_X_–N volatilised) ^–1^) | 0.002 | 0.018 | 0.010 | IPCC, 2019 |
|  | *Frac_GASF1_* | fraction of urea-N that volatilises as NH_3_ and NO_x_ under different conditions *i* (kg N volatilised (kg of N applied)^-1^) | 0.03 | 0.43 | 0.15 | IPCC, 2019 |
|  | *Frac_GASF2_* | fraction of NPK compound-N that volatilises as NH_3_ and NO_x_ under different conditions *i* (kg N volatilised (kg of N applied)^-1^) | 0.02 | 0.33 | 0.11 | IPCC, 2020 |
|  | *EF_5_* | emission factor for N_2_O emissions from N leaching and runoff (kg N_2_O–N (kg N leached and runoff) ^–1^) | 0 | 0.02 | 0.01 | IPCC, 2019 |
|  | *Frac_LEACH-(H)_* | fraction of all N added to/mineralised in managed soils in regions where leaching/runoff occurs that is lost through leaching and runoff (kg N (kg of N additions) ^–1^) | 0.01 | 0.73 | 0.24 | IPCC, 2019 |

^a^ Database used in this study include the Chinese Life Cycle Database (CLCD v0.8; <https://efootprint.net/login>) and the Swiss Ecoinvent 2.2 database (<https://simapro.com/databases/ecoinvent/>). The description of the face-to-face field investigation was shown in the Section 2.2.1.

**Table B** Selection of input parameters of carbon footprint evaluation model of organic rice production mode.

| Category | Parameters | Description | Lower boundary | Upper boundary | Initial value | Source ^a^ |
| --- | --- | --- | --- | --- | --- | --- |
| Background parameters | *EF_fym_* | Emission factor of farm yard manure (kg CO_2_ kg^–1^) | 0.0059 | 0.0073 | 0.0066 | Database |
|  | *EF_elec_* | Emission factor of electricity consumption (kg CO_2_ kWh^–1^) | 1.14 | 1.40 | 1.27 | Database |
|  | *EF_dies_* | Emission factor of diesel consumption (kg CO_2_ kg^–1^) | 0.33 | 0.41 | 0.37 | Database |
|  | *EF_rs_* | Emission factor of rice seed (kg CO_2_ kg^–1^) | 1.69 | 2.07 | 1.88 | Database |
| Activity data | *A_fym_* | Amount of farm yard manure (kg ha^–1^) | 10368 | 15552 | 12960 | Investigation |
|  | *C_elec_* | Consumption of electricity (kWh ha^–1^) | 355 | 533 | 444 | Investigation |
|  | *C_dies_* | Consumption of diesel (kg ha^–1^) | 50 | 76 | 63 | Investigation |
|  | *A_rs_* | Amount of rice seed (kg ha^–1^) | 46 | 68 | 57 | Investigation |
|  | *Y* | Yield of rice (kg ha^–1^) | 5340 | 8010 | 6675 | Investigation |
| CH_4_ estimation parameters | *EF_C_* | Baseline emission factor for continuously flooded fields without organic amendments (kg CH_4_ ha^–1^ day^–1^) | 0.89 | 1.96 | 1.32 | IPCC, 2019 |
|  | *t* | Cultivation period of rice (d) | 73 | 147 | 118 | Investigation |
|  | *SF_w_* | Scaling factor to account for the differences in water regime during the cultivation period (–) | 0.53 | 0.94 | 0.71 | IPCC, 2019 |
|  | *SF_P_* | Scaling factor to account for the differences in water regime in the pre-season before the cultivation period (–) | 0.80 | 0.99 | 0.89 | IPCC, 2019 |
|  | *CFOA_s_* | Conversion factor for straw (Straw incorporated >30 days before cultivation) | 0.11 | 0.28 | 0.19 | IPCC, 2019 |
|  | *CFOA_fym_* | Conversion factor for farm yard manure | 0.15 | 0.28 | 0.21 | IPCC, 2019 |
|  | *Ex* | The exponent in Equation 5.3 | 0.54 | 0.64 | 0.59 | IPCC, 2019 |
| N_2_O estimation parameter | *EF_1FR_* | Emission factor for N_2_O emissions from N inputs to flooded rice (single and multiple drainage) (kg N_2_O-N (kg N input)^–1^) | 0 | 0.016 | 0.005 | IPCC, 2019 |
|  | *EF_4_* | Emission factor for N_2_O emissions from atmospheric deposition of N on soils and water surfaces (kg N–N_2_O (kg NH_3_–N + NO_X_–N volatilised) ^–1^) | 0.002 | 0.018 | 0.010 | IPCC, 2019 |
|  | *Frac_GASM_* | fraction of applied organic N fertiliser materials (*F_ON_*) and of urine and dung N deposited by grazing animals (*F_PRP_*) that volatilises as NH_3_ and NO_X_, kg N volatilised (kg of N applied or deposited) ^–1^ | 0 | 0.31 | 0.21 | IPCC, 2019 |
|  | *EF_5_* | emission factor for N_2_O emissions from N leaching and runoff (kg N_2_O–N (kg N leached and runoff) ^–1^) | 0 | 0.020 | 0.011 | IPCC, 2019 |
|  | *Frac_LEACH-(H)_* | fraction of all N added to/mineralised in managed soils in regions where leaching/runoff occurs that is lost through leaching and runoff (kg N (kg of N additions) ^–1^) | 0.01 | 0.73 | 0.24 | IPCC, 2019 |

^a^ Database used in this study include the Chinese Life Cycle Database (CLCD v0.8; <https://efootprint.net/login>) and the Swiss Ecoinvent 2.2 database (<https://simapro.com/databases/ecoinvent/>). The description of the face-to-face field investigation was shown in the Section 2.2.1.
